# Management of Severe Multifactorial Eyelid Ectropion With Lateral Tarsal Strip Procedure and Full-Thickness Skin Graft

**DOI:** 10.7759/cureus.23462

**Published:** 2022-03-24

**Authors:** Abelardo Medina

**Affiliations:** 1 Surgery, University of Mississippi Medical Center, Jackson, USA

**Keywords:** radiation, facial palsy, skin graft, lateral tarsal strip, ectropion

## Abstract

Secondary ectropion is a relatively common anatomo-functional abnormality of the lower eyelids. They can be seen in numerous clinical settings and generate disabling symptoms and even severe complications such as corneal ulceration, globe perforation, and blindness. Based on their etiology, cases of secondary ectropion are grouped into involutional, paralytic, cicatricial, and mechanical.

Multiple techniques have been developed to treat specific abnormalities associated with this condition. Therefore, a comprehensive local assessment is crucial to select one or more surgical procedures for each particular situation.

This report describes a patient with multiple contributing factors for severe ectropion of left lower eyelid treated with lateral tarsal strip procedure and full-thickness skin graft. Other surgical options and their indications are briefly reviewed.

## Introduction

Ectropion is defined as the eversion of the eyelid margin resulting in its lack of contact with the globe. This abnormal condition can lead to chronic conjunctival inflammation or infection, photophobia, eye dryness, local pain, decreased vision, keratopathy, and epiphora [1]. Unfortunately, untreated cases may also induce severe complications such as corneal ulceration, globe perforation, and blindness [2]. Secondary or acquired causes of ectropion are divided into four categories: involutional, paralytic, cicatricial, and mechanical. Involutional ectropion, the most frequent variant, is produced by age-related horizontal laxity of the orbicularis muscle and/or canthal tendons (medial and/or lateral). The disinsertion of the lower eyelid retractors may also play a role in some cases [3]. Paralytic ectropion can be developed after the loss of tone of the orbicularis oculi muscle (i.e., facial nerve paralysis, stroke, etc.). The cicatricial type characteristically occurs when scar contractures generate vertical shortening of the anterior (skin and orbicularis oculi muscle) and/or middle lamella (tarsal plate or orbital septum). Causes of cicatricial ectropion include transcutaneous blepharoplasty, Mohs surgery, and trauma, among others. Finally, mechanical ectropion is mainly produced by the mass effect of a tumor.

Even though several procedures have been described for ectropion repair and they should be tailored according to the specific etiology and local condition, the lateral tarsal strip (LTS) with or without adjuvant procedures (i.e., full-thickness skin graft, medial spindle canthoplasty, others) is a relatively simple and well-established horizontal lid tightening technique to restore the tone of the lower eyelid and anatomic position of the lateral canthus with reliable outcomes among surgeons [1,4]. This report describes the clinical course and surgical treatment for a severe ectropion in a patient with multiple contributing factors and underlying comorbidities. Other common surgical techniques and their main indications are also reviewed.

## Case presentation

An 80-year-old male with a history of skin cancer of the left temporal/mastoid region was treated with extensive resection (including craniotomy) and high-dose radiation therapy. Subsequently, the patient underwent multiple surgical procedures to manage radiation-induced injuries to the left side of the face and temporal bone. These procedures included anterolateral thigh free flap, cervicofacial flap, pectoral major muscle flap and split-thickness skin graft (STSG), debridement of left mastoid and middle ear cavities, among others. In addition, an extensive subcutaneous dissection was performed from the suprahyoid region to the temporal region reaching anteriorly the lateral canthus and nasolabial fold.

Three years later, the patient was referred to our clinic by an ophthalmologist requesting correction of the left ectropion prior to a planned cataract surgery. He had significant comorbidities including atrial fibrillation, chronic left otitis externa and temporal bone osteomyelitis, non-insulin-dependent diabetes mellitus (NIDDM), hypertension and history of seizures, syncope, and transient ischemic attack (TIA); no tobacco or alcohol consumption.

**Figure 1:**
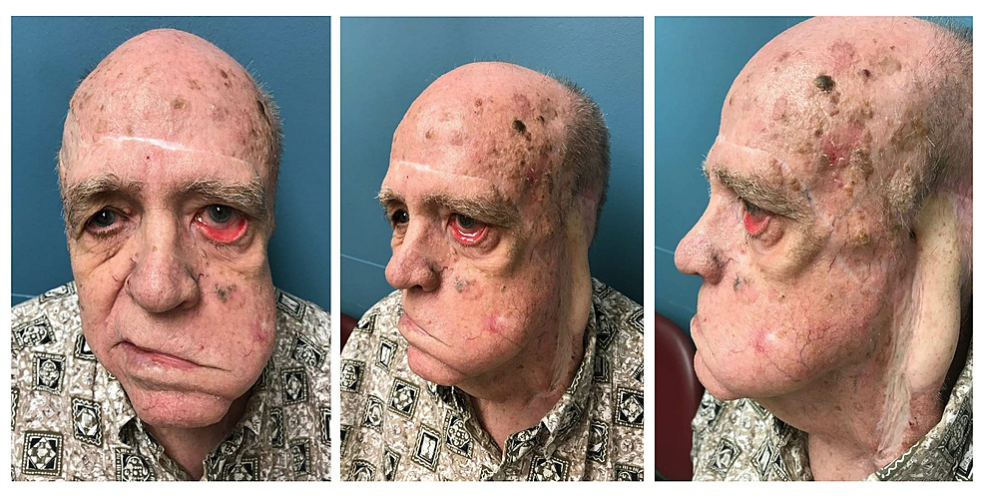
Preoperative clinical condition An 80-year-old male presented with severe left lower eyelid ectropion, a history of extensive excision of skin cancer with multiple reconstructive surgeries, left facial nerve palsy, and radiation. The patient complained of inability to close eyelids, eye dryness, itching sensation, and permanent epiphora. On physical examination, he presented wide exposure of inferior conjunctival fornix and caruncle as well as signs of chronic conjunctivitis such as conjunctival hyperemia, papillary hypertrophy, and presence of keratinization without stenosis of the lacrimal punctum. Of note, he presented significant scars along the forehead, left temple, and adjacent parotid region. In addition, he had a large area of the right scalp previously resurfaced with split-thickness skin graft over galea.

During our initial assessment, the patient complained of inability to close eyelids, eye dryness, local itching sensation, and permanent epiphora. On physical examination, he presented a left-sided facial nerve paralysis and severe left lower eyelid ectropion associated with blurry vision, wide exposure of the inferior conjunctival fornix and caruncle as well as signs of chronic conjunctivitis such as conjunctival hyperemia, papillary hypertrophy, and presence of keratinization along the mucocutaneous junction of the posterior lamella without stenosis of the lacrimal punctum. Bilateral extraocular movements (EOM) and corneal surfaces were normal. In addition, increased soft tissue fibrosis of the left cheek and skin deficit on the left lower eyelid were noticed. The patient also presented significant scars along the forehead, left temple, and adjacent parotid region. Furthermore, he had a large area of the right scalp previously resurfaced with split-thickness skin graft over galea (Figure 1).

**Figure 2:**
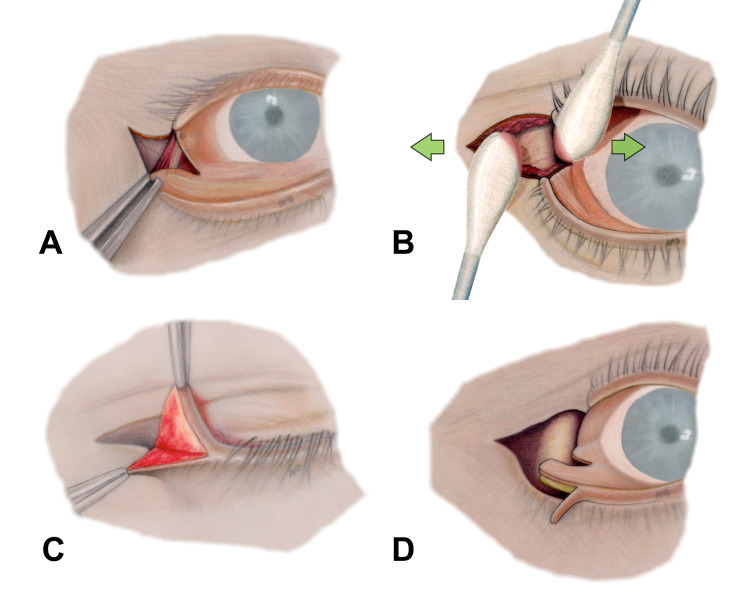
Lateral tarsal strip procedure (A) Lateral canthotomy followed by inferior cantholysis. (B) Exposure of lateral orbital rim by stretching overlying soft tissues between two cotton swabs. (C) An incision along the gray line followed by dissection between anterior and posterior lamellae. (D) Excision of mucocutaneous lining along the superior border of the tarsal plate followed by a conjunctival incision along its inferior border.

Based on clinical findings, a lateral tarsal strip with or without adjuvant procedures was planned to restore the eyelid anatomy and functions. Due to the patient’s comorbidities, the surgery was performed under general anesthesia. At this point, the estimated length of the LTS was marked on the skin after pulling the lower eyelid laterally to reach proper tightness. Next, local infiltration of 3 ml of 1% lidocaine with 1: 200,000 epinephrine was applied within the left lateral canthus and upper and lower eyelids. The lateral canthotomy started with a transverse 1-cm incision of the skin on the lateral canthus followed by incision of the corresponding conjunctiva. The dissection in-between these two incisions was slightly angled down to avoid damage of the superior crura of the lateral canthal tendon. The lower eyelid was fully mobilized after the completion of an inferior cantholysis with curved Westcott scissors (Figure 2A). While protecting the orbital portion of the orbicularis oculi muscle away from the lateral canthus, the nearby orbital rim was exposed by stretching overlying soft tissues between two cotton swabs (Figure 2B).

**Figure 3:**
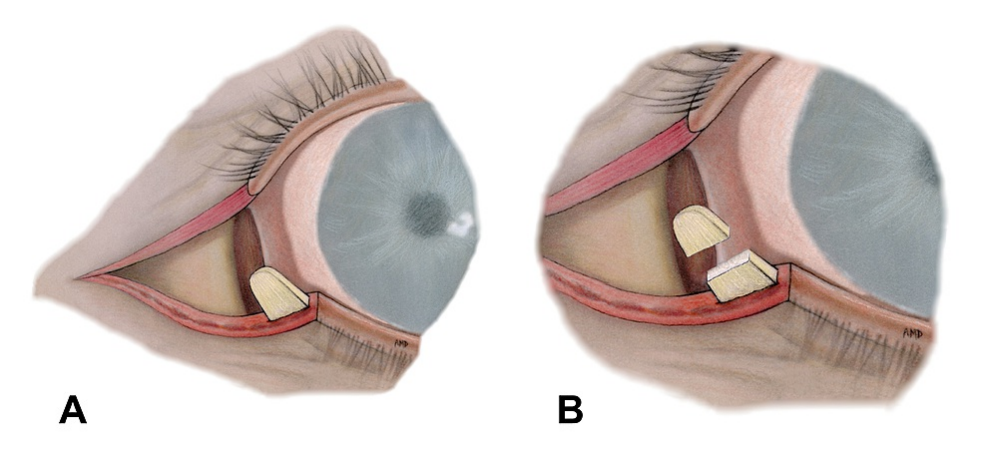
Lateral tarsal strip procedure, cont’d. (A) Conjunctival lining of the tarsus is charred with bipolar electrocautery followed by scraping with a No. 15 blade to complete the tarsal strip isolation. (B) After checking the anticipated position of the new lateral commissure and appropriate tightness of the eyelid, redundant canthal tendon and distal tarsus are trimmed.

Subsequently, separation of anterior (skin and orbicularis muscle) and posterior lamellae (tarsus and conjunctiva) of the eyelid was carried out. By holding the lateral end of the tarsal plate, an incision along the gray line was made with Westcott scissors followed by dissection of a pocket between the orbicularis muscle and tarsal plate (Figure 2C). Then, the mucocutaneous lining along the superior border of the tarsal plate was sharply excised. The tarsal strip was further released by incising the conjunctiva along its lower border (Figure 2D). Thereafter, to complete the tarsal strip isolation, the conjunctival lining of the tarsus was charred with bipolar electrocautery followed by scraping with a No. 15 blade (Figure 3A).

**Figure 4:**
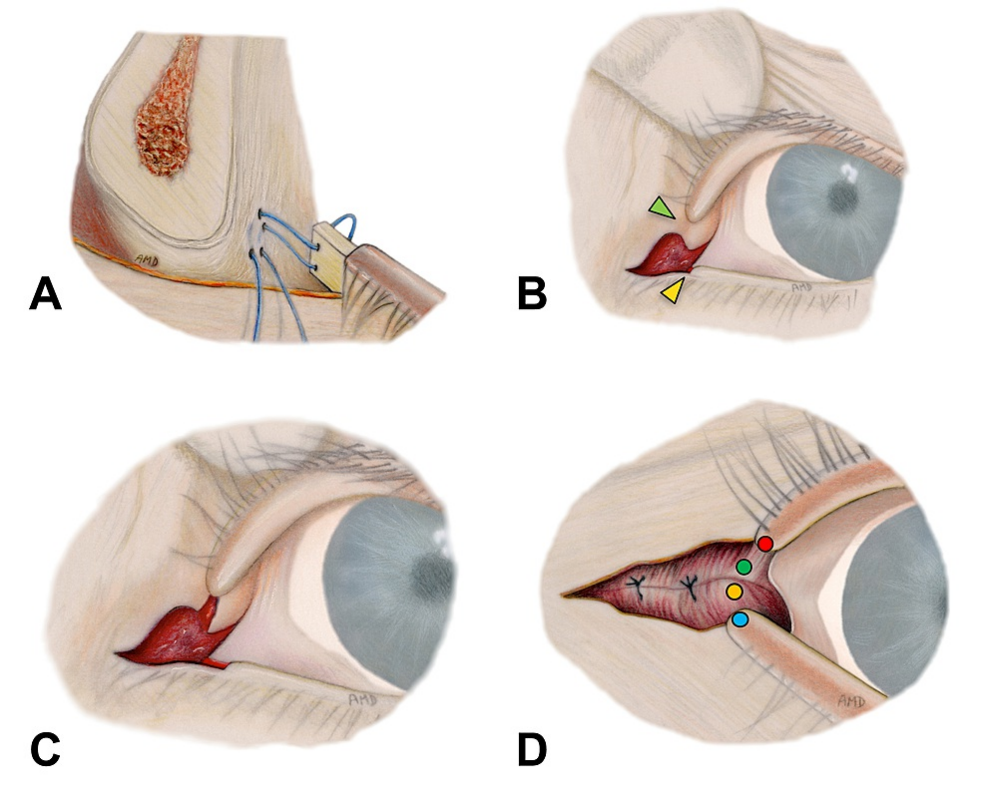
Lateral tarsal strip procedure, cont’d (A) Tarsal strip anchoring to the periosteum of the lateral orbital rim from inside outward. (B-C) Reconstruction of a sharp lateral canthal angle starting with the removal of existing epithelium between the superior cut edge of the canthotomy and the adjacent lateral end of the upper tarsus - green arrowhead. A small amount of epithelium at the lower eyelid was also removed to line up de-epithelialized edges - yellow arrowhead. (D) Single buried knot 6-0 vicryl stitch between the end of the upper tarsus and its lower counterpart. The needle is passed through the deep tissue inside lateral canthotomy (green circle) to exit at the gray line of the upper eyelid adjacent to the beginning of the de-epithelialized area (red circle), and then from the gray line of the lower eyelid (blue circle) to its corresponding deeper tissue (yellow circle).

After checking the anticipated position of the new lateral commissure and appropriate tightness of the eyelid, the redundant canthal tendon and distal tarsus were trimmed (Figure 3B). A 5-0 Prolene with a double-armed half-circle curved needle was used to anchor the tarsal strip. The two needles were passed from the posterior to the anterior surface of the tarsal strip near its corners making sure to have at least 1-mm of tissue around. The strip was then anchored to the periosteum of the lateral orbital rim (at the level of Whitnall’s tubercle) from inside outward. To accomplish this, the first needle was slid backward for a few millimeters over the surface of the inner aspect of the lateral orbital rim to pierce the periosteum in a posterior to anterior direction. Similarly, the second needle grabbed the periosteum 2 mm below the first one (Figure 4A).

The suture was tied off with several knots after the tarsal strip obtained adequate side-to-side apposition over the bony surface. Both suture threads were buried under the soft tissue in a posterior direction and finally cut at the resulting exit points. Next, disrupted fibres of orbicularis muscle were repaired with interrupted stitches of 6-0 vicryl. The reconstruction of a sharp lateral canthal angle began with the removal of the existing epithelium between the superior cut edge of the canthotomy and the adjacent lateral end of the upper tarsus (Figure 4B, green arrowhead; Figure 4C). A small amount of epithelium at the lower eyelid was also removed to line up de-epithelialized edges (Figure 4B, yellow arrowhead; Figure 4C). Thereafter, the end of the upper tarsus and its lower counterpart were approximated with a single buried knot 6-0 vicryl stitch (Figure 4D). Thus, the needle was passed through the deep tissue at the superior cut edge of the lateral canthotomy (Figure 4D; green circle) to exit at the gray line of the upper eyelid adjacent to the de-epithelialized area (Figure 4D; red circle), and then from the gray line of the lower eyelid (Figure 4D; blue circle) to its corresponding deep tissue (Figure 4D; yellow circle). This stitch provided further support to the lower eyelid.

**Figure 5:**
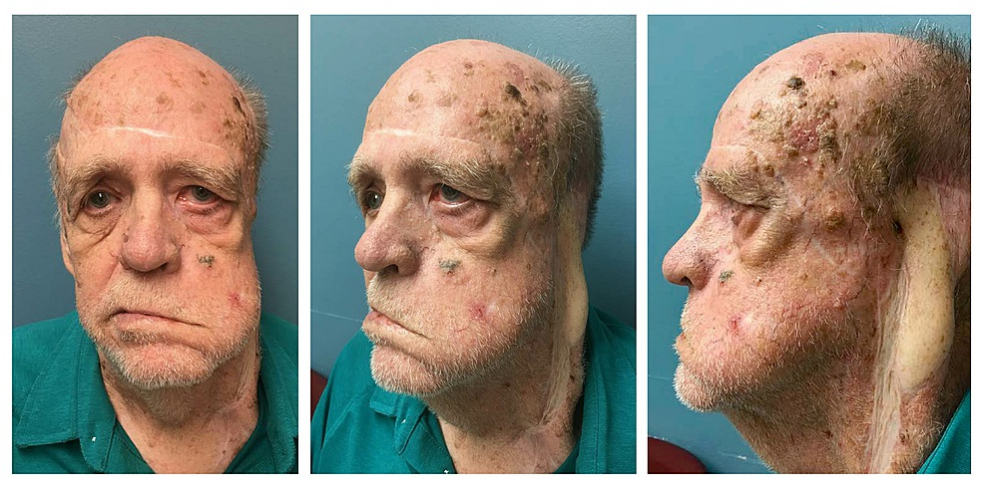
Long-term postoperative outcome after lateral tarsal strip (LTS) and full-thickness skin graft (FTSG) Horizontal shortening (LTS) and vertical lengthening (FTSG) leading to significant improvement of the lower eyelid ectropion and punctal position. Most of his preoperative cosmetic concerns and presenting symptoms were reversed. During the long-term assessment, the patient did not complain of blurry vision, epiphora or local itchiness. He reported only occasional mild sensation of eye dryness, which he assigned a value of 1 in a scale of 1 to 10 (where 10 corresponds to eye dryness that he had prior to the surgery).

As the skin of the lower eyelid was deficient, only a narrow eyelash follicle-containing portion was excised at the level of the LTS. Next, a subciliary incision was made from this point up to near the medial canthal angle. Thereafter, a dissection under the orbicularis muscle was made in a downward direction. This dissection created a well-vascularized wound bed for the application of a full-thickness skin graft (FTSG) previously harvested from the right upper eyelid. The FTSG was slightly oversized with respect to the wound bed dimensions (2.5x1 cm) in order to prevent ectropion recurrence. This skin graft was secured in place with 5-0 fast-absorbing gut sutures and subsequently covered with a tie-over bolster dressing. Finally, the canthal extension of the incision was closed in two layers. The deeper tissues were approximated with interrupted stitches of 6-0 vicryl. The skin, starting at the newly established lateral end of the lashline, was closed with interrupted stitches of 5-0 Prolene. At the end of the case, the keratinized conjunctiva along the lid margin was carefully removed with bipolar electrocautery. The patient was sent home with bacitracin ointment to apply on lateral canthotomy incision and 0.3% tobramycin ophthalmic solution to apply into both eyes. The skin stitches were removed five days after surgery and bolster dressing was discontinued at day 10. The patient did not experience postoperative complications. During a long-term follow-up, the patient exhibited adequate eyelid contact with the globe and punctum position. Even though minimal scleral exposure was noticed inferiorly, the lower eyelid had normal tension with a significant increase in the distance between the lid margin and inferior orbital rim/tear trough level (Figure 5). The patient underwent cataract surgery five months later without complications.

## Discussion

Ectropion is one of the most common clinical conditions affecting lower eyelids [1]. Apart from congenital presentations, acquired ectropion cases are grouped into four categories namely involutional, paralytic, cicatricial, and mechanical.

Numerous surgical procedures have been described to treat horizontal laxity and/or vertical deficit of the lower eyelid. In mild cases associated with cosmetic procedures, canthopexy is a reasonable solution since it can be done through blepharoplasty incisions with minimal extra tissue dissection and operative time [5]. Full-thickness eyelid resection at different locations has been used to correct the lid malposition and lack of tone [6]. However, wedge excisions can induce untoward results such as lid margin notching, scar contracture of anterior and/or posterior lamella, phimosis, and ectropion recurrence [7]. Alternatively, a vertical flap can be harvested from the same redundant eyelid segment and subsequently inset in a horizontal position at the level of the lid-cheek junction [8]. This procedure produces a combination of horizontal shortening and vertical lengthening of the eyelid. Similar aim is achieved with lateral eyelid-block excision with canthoplasty and FTSG. This technique develops a horizontal myocutaneous flap along the entire lower eyelid margin [9]. The flap is advanced laterally until the appropriate lid tone is restored. Then, the lateral end of the flap is reattached to the lateral canthal ligament followed by the use of FTSG to manage the shortage of eyelid skin [9]. Interestingly, the Bick’s procedure or resection of the lateral aspect of the lower eyelid without disrupting the lateral canthal tendon appeared as a quick and easy surgical refinement to exclusively manage horizontal laxity. Unfortunately, the Bick’s procedure seems to have a higher risk of rounding and/or medial displacement of the lateral canthal angle [10]. For patients with medial ectropion and punctum eversion, medial spindle canthoplasty is a relatively simple and effective technique in which a retropunctal diamond excision is performed. Through this window, 6-0 vicryl sutures are used to reattach the lower lid retractor to the tarsal plate and conjunctiva [11]. Vertical lid tightening can also be achieved by reattachment or plication of the lower eyelid retractor to the inferior tarsal border via transconjunctival or transcutaneous approach [1,3]. A medial canthopexy with bone anchoring system could be another valid option [12]. For punctal keratinization or stenosis, a snip punctoplasty with posterior ampullectomy will secure appropriate drainage into the canaliculus.

For mild cases of cicatricial ectropion secondary to scar contracture, the scar release associated with Z-plasty or V-Y advancement flaps may be enough to correct the eyelid malposition. In addition, rotational flaps of skin only, from upper to lower eyelids with either medial or lateral blood supply, have been extensively used in clinical practice. The eyelid reconstruction with myocutaneous flaps is reserved for severe cicatricial ectropion where periosteal or fascial suspension sutures should be incorporated to minimize downward secondary lid retraction [6,8]. Unfortunately, in general, they cannot fully recreate the appearance, pliability, and ultimately the subtle functions of the native eyelid skin and have risks of lagophthalmos and bulging deformity [6].

LTS is perhaps the most popular and repeatedly used surgical procedure to manage significant ectropion in a large variety of clinical settings [7,13]. This technique, performed under local or general anesthesia, provides not only reliable outcomes among surgeons, but also high satisfaction among patients. In severe cases, the use of double suture and conjunctival cuts in the LTS have been described to decrease the recurrence rate [14]. Thus, in the described patient, LTS was utilized to correct a multifactorial, long-lasting, and severe eversion of the eyelid margin and punctum (Figure 1). By producing horizontal shortening, this procedure enhanced the eyelid tone and anatomic position of the lateral canthus [1]. In addition, by de-epithelializing, the mucocutaneous bridge between the lateral end of the upper tarsus and upper cut edge of the lateral canthotomy helps establish a sharp lateral canthal angle while preventing the upper lid overhang and lower lid imbrication (Figure 4B-4C) [7]. Unfortunately, LTS did not fully reverse the patient’s eyelid eversion and punctum ectopic location (Figure 1). As described above, the patient exhibited involutional age-related changes and complete facial nerve paralysis that markedly contributed to the soft tissue laxity and muscular hypotonic condition. The patient also underwent multiple surgical interventions and high-dose radiation, which in turn inevitably induced significant multi-layered soft tissue fibrosis and scar contracture. Furthermore, the extensive facial dissection performed in previous reconstructive procedures presumably disrupted retaining cutaneous ligaments within zygomatic, maxillary, and masseteric areas [15]. Taken together, the excessive soft tissue laxity, pulling forces from aforementioned contributing factors along with gravity and deficit of eyelid skin could explain the incomplete restoration of the ectropion with LTS alone. Accordingly, FTSG from the right upper eyelid was added on the subciliary area to address the vertical skin shortage, and thus, to accomplish not only appropriate contact of the lid margin with the globe, but also adequate position of the punctum [10]. In general, FTSG has a high rate of graft take, even in previously irradiated fields [16]. The palpebral source for FTSG harvest was chosen taking into account skin redundancy of the patient’s upper eyelids, its unquestionable match in color, texture and thickness with the skin of the lower eyelid, and the excellent cosmetic result at the donor site. Skin grafts from other sites (i.e., retroauricular, supraclavicular, etc.) can also be considered for anterior lamella reconstruction. In cases of insufficient support of the lid margin, auricular chondrocutaneous composite grafts are acceptable options [17]. Fascial sling is another available solution, especially in cases of severe post-burn ectropion [18].

In the reported patient, a mid-face lift could facilitate the recruitment of skin upward to help with the ectropion correction [19,20]. Through minimal additional incisions, a supra- or sub-periosteal dissection of the malar tissues can be accomplished to subsequently perform their elevation and re-attachment with or without lateral orbicularis oculi muscle suspension [19,20]. However, the presence of multiple underlying comorbidities and substantial subcutaneous dissection in previous surgery disregarded this option from consideration. Similarly, even though the patient had bilateral brow ptosis, no correction was planned (i.e., suprabrow lift) since he had considerable forehead and left temple scars and no visual field impairment (Figure 1).

In summary, the reported patient underwent horizontal shortening in combination with vertical lengthening of the anterior lamella. These procedures led to significant improvement of his left lower eyelid ectropion and punctal position. The area of FTSG reached an excellent match with the surrounding skin and without shrunken appearance. Most of his preoperative cosmetic concerns and presenting symptoms of ocular irritation were also reversed. During a long-term assessment (32 months after surgery), the patient did not complain of blurry vision, epiphora, and local itchiness. He has not experienced any episode of conjunctivitis or corneal ulceration. He reported only an occasional mild sensation of eye dryness, which he assigned a value of 1 on a scale of 1 to 10 (where 10 corresponds to the patient’s sensation of eye dryness prior to the surgery).

## Conclusions

The management of severe lower eyelid ectropion requires a comprehensive knowledge of the loco-regional anatomy and an accurate preoperative assessment. This evaluation needs to take into account all contributing factors that may play a role affecting different components (lamellae) of this complex anatomical structure. Accordingly, the subsequent surgical plan has to be patient-specific and may consist of one or more techniques.
